# Aquaporin-4 as a cerebrospinal fluid biomarker of Alzheimer’s disease

**DOI:** 10.1186/s40035-024-00451-8

**Published:** 2024-11-29

**Authors:** Nerea Gómez de San José, Steffen Halbgebauer, Petra Steinacker, Sarah Anderl-Straub, Samir Abu-Rumeileh, Lorenzo Barba, Patrick Oeckl, Giovanni Bellomo, Lorenzo Gaetani, Andrea Toja, Sára Mravinacová, Sofia Bergström, Anna Månberg, Alberto Grassini, Innocenzo Rainero, Peter Nilsson, Lucilla Parnetti, Markus Otto

**Affiliations:** 1 Department of Neurology, Ulm University Hospital, Albert-Einstein-Allee 23, 89081 Ulm, Germany; 2 Department of Neurology, University Clinic, University Hospital Halle, Martin Luther University, Ernst-Grube Strasse 40, 06120 Halle (Saale), Germany; 3 Section of Neurology, Department of Medicine and Surgery, University of Perugia, Piazza dell’Università, 1, 06123 Perugia, Italy; 4 Department of Neuroscience, “Rita Levi Montalcini”, University of Torino, Via Cherasco 15, 10126 Turin, Italy; 5 Department of Protein Science, SciLifeLab, KTH Royal Institute of Technology, Brinellvägen 8, 114 28 Stockholm, Sweden; 6 German Center for Neurodegenerative Diseases (DZNE), Oberer Eselsberg 45, 89081 Ulm, Germany

The diagnosis of Alzheimer’s disease (AD) relies on the clinical evaluation of patients, often complemented by the analysis of core cerebrospinal fluid (CSF) biomarkers (Aβ42/40, phosphorylated-Tau and total-tau) [1]. However, it is clear nowadays that alterations other than Aβ and tau deposition, e.g., blood–brain-barrier (BBB) impairment [2] and impaired protein clearance [3], may take place in early disease stages, before consistent neurodegeneration occurs. Therefore, additional CSF biomarkers are needed.

Aquaporin-4 (AQP4) is a water channel highly expressed in the central nervous system, prominently enriched in the perivascular endfeet of astrocytes wrapping around blood vessels [4]. It plays a crucial role in the glymphatic system, enabling the exchange between CSF and interstitial fluid and supporting the clearance of brain solutes. The impact of AQP4-mediated glymphatic flow on the clearance of solutes may hold clinical relevance, particularly in AD [5]. However, no well validated AQP4 immunoassay is available for the analysis of protein concentrations in CSF.

The study aimed to analyze CSF AQP4 levels, using a newly developed homemade enzyme-linked immunosorbent assay (ELISA) in a discovery cohort of 157 CSF samples gathered at Ulm University Hospital. The cohort comprised 40 AD, 21 primary progressive aphasia (PPA), 20 behavioral variant frontotemporal dementia, 17 amyotrophic lateral sclerosis (ALS), 21 Lewy body disease (LBD) and 38 controls (CON) (Table S1). To reinforce the robustness of the results, a validation step using two independent cohorts from University of Perugia (validation cohort I) and University of Turin (validation cohort II) was conducted. Those cohorts comprised a total of 176 additional CSF samples: 14 preclinical AD (preAD), 51 mild cognitive impairment due to AD (AD-MCI), 39 AD-dementia (ADD) patients, 17 non-AD MCI and 55 CON (Table S2). Diagnosis of the CON participants is described in Table S3.

The homemade ELISA assay successfully passed all the analytical validation tests for precision, parallelism, spike-recovery, dilutional linearity, and protein stability as well as a comparison to an antibody-based suspension bead array assay (Tables S4 and S5, Figs. S1S2, S3). 

A multiple linear regression model was fitted to estimate the relationship between the clinical diagnosis (predictor) and CSF AQP4 concentration (outcome) while accounting for age and sex. Among the covariates analyzed, only age (log-transformed β_age_ = 0.013, *P* < 0.001) (Table S6 and Fig. S4) exhibited a significant association with CSF AQP4.

In the discovery cohort post-hoc analysis (Tukey’s Test) indicated higher AQP4 concentration in AD patients than in CON (*P* < 0.001), ALS (*P* = 0.015), and LBD (*P* = 0.012) patients (Fig. 1a). Moreover, ROC analysis was conducted to evaluate the diagnostic performance of CSF AQP4 in distinguishing AD patients from CON. The area under the curve (AUC) for the AD vs. CON discrimination was 0.81 (95% CI: 0.71–0.90, *P* < 0.001) (Fig. 1b).

**Fig. 1:**
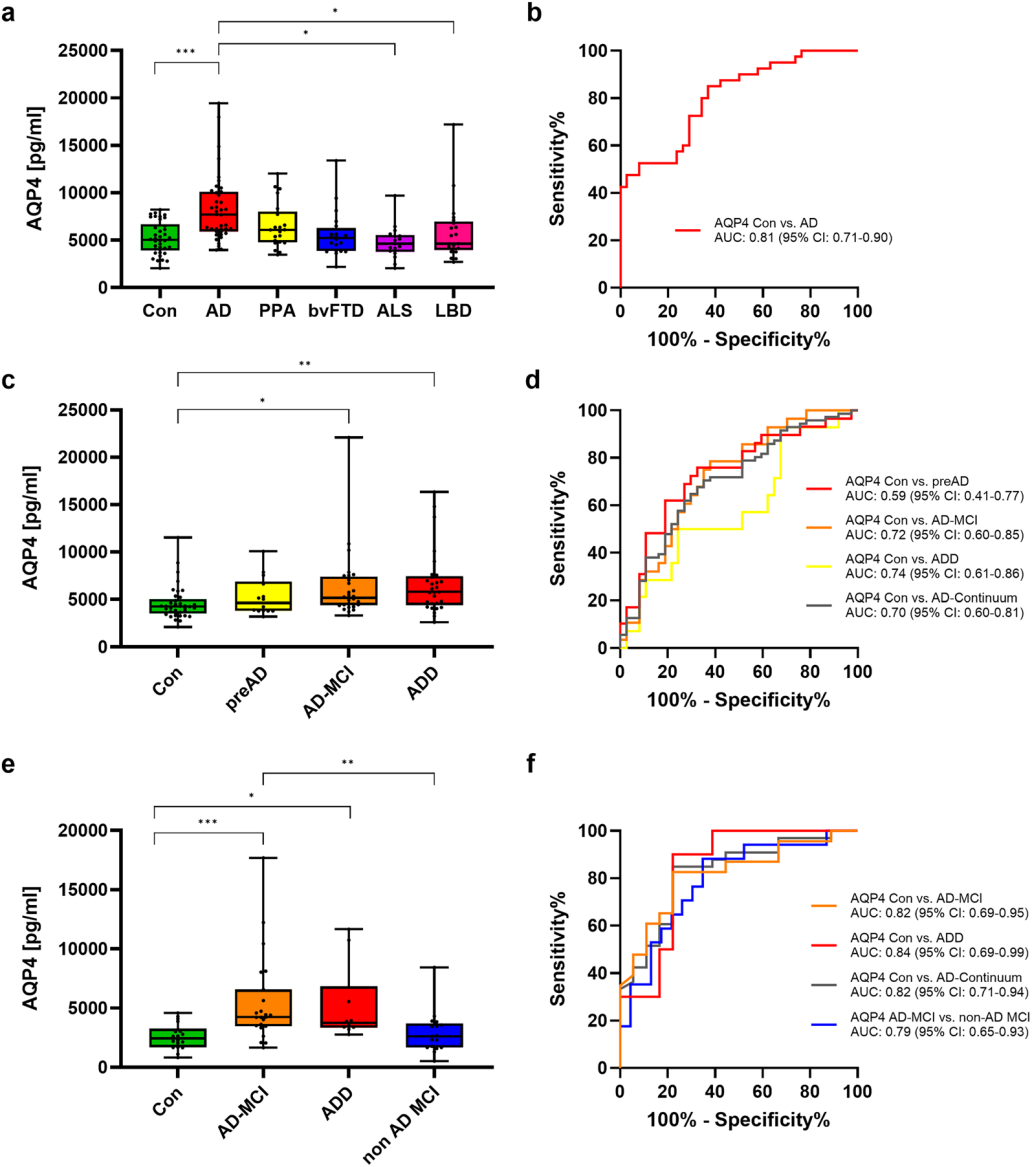
Aquaporin 4 (AQP4) CSF analysis in three different cohorts. CSF AQP4 in the discovery cohort (*n* = 157) including 38 CON, 40 AD, 21 PPA, 20 bvFTD, 17 ALS and 21 LBD (**a, b**) and in the validation cohorts I and II: cohort I (*n* = 108) including 37 CON, 14 preAD, 28 AD-MCI and 29 ADD (**c, d**) and cohort II (*n* = 68) including 18 CON, 23 AD-MCI, 10 ADD and 17 non-AD MCI (**e, f**). Boxplots represent median, Q1 and Q3 quartiles, and whiskers from minimum to maximum. *P < 0.05, **P < 0.01, and ***P < 0.001. **b, d, f** Receiver operating characteristics curve analysis of CSF AQP4. AD, Alzheimer’s disease; ALS; amyotrophic lateral sclerosis; AUC, area under the curve; bvFTD, behavioral variant frontotemporal dementia; CON, control; CSF, cerebrospinal fluid; IQR, interquartile range; LBD, Lewy body disease; PPA, primary progressive aphasia

To validate the findings within AD, the CSF levels of AQP4 were further measured in two independent validation cohorts (Table S2). 

Validation cohort I included patients with preAD, AD-MCI and ADD in addition to CON subjects. The univariate analyses revealed higher AQP4 protein concentrations in CSF of AD-MCI (*P* = 0.011) and ADD (*P* = 0.002) patients than in CON (Fig. 1c). The ROC analysis revealed the best performance for the CON versus ADD comparison with an AUC of 0.74 (95% CI 0.61–0.86, *P* < 0.001) (Fig. 1d).

Validation cohort II included individuals with AD-MCI, ADD, CON, in addition to a group of non-AD MCI participants. Significantly increased levels of CSF AQP4 were observed in the AD-MCI (*P* < 0.001) and ADD (*P* = 0.028) patients compared to CON (Fig. 1e). In addition, the individuals with AD-MCI had significantly higher levels than those with non-AD MCI (*P* = 0.004) (Fig. 1e). Furthermore, the ROC analyses revealed the following AUCs: 0.82 (CON vs. AD continuum) (95% CI 0.71–0.94, *P* < 0.001), 0.82 (CON vs AD-MCI) (95% CI 0.69–0.95, *P* < 0.001), 0.84 (CON vs. ADD) (95% CI 0.69–0.99, *P* = 0.003) and 0.79 (AD-MCI vs. non-AD MCI) (95% CI 0.65–0.93, *P* = 0.002) (Fig. 1f).

Subsequently, the association between AQP4 and the core biomarkers t-tau and p-tau was investigated among individuals with AD, regardless of their disease stage (preAD, AD-MCI or ADD), across the three cohorts under analysis (Fig. S5). Notably, in the discovery cohort, a moderate-to-strong correlation between AQP4 and both p-tau (*r* = 0.7, 95% CI 0.35–0.87, *P* < 0.001) and t-tau (*r* = 0.75, 95% CI 0.44–0.90, *P* < 0.001) was observed in AD patients (Fig. S5a). This correlation persisted across the entire cohort (Fig. S5b). Similar trends, although with comparatively weaker correlations, were delineated in both validation Cohort I and Cohort II (Fig. S5c–f). Only a larger sample size can disclose the true strength of this association.

Furthermore, we assessed the association between CSF AQP4 levels and cognitive decline (mini-mental state examination). We found negative spearman *r* values in all three cohorts, but the correlation was significant only in validation cohort II with an *r* value of − 0.48 (− 0.65 to − 0.27) (*P* < 0.0001) (Table S7).

The successfully developed and analytically validated ELISA revealed higher concentrations of AQP4 in AD than in control patients, a pattern replicated in two validation cohorts. These findings supported the previously published data using a semi-quantitative antibody-based suspension bead array assay [6]. To our knowledge, this is the first immunoassay able to perform absolute AQP4 quantification in CSF samples. A recent publication using a commercial AQP4 ELISA reported increased AQP4 levels in the CSF of AD patients [7]. However, we were unable to identify any signal in CSF using this commercial assay.

Elevated AQP4 levels in individuals with MCI were found, specific to AD as previously described by Bergström et al. in MCI patients with abnormal tau levels [6].

Altered AQP4 polarization is noted in the frontal and temporal cortex of AD patients. Elevated CSF AQP4 thereby may compensate for the loss in astrocytic endfeet, aiming to restore glymphatic flow [8]. Additionally, elevated CSF AQP4 levels in AD patients might be linked to changes in astrocytic reactivity, a characteristic of neurodegenerative diseases.

The potential of AQP4 as an early biomarker may arise from the failure of the glymphatic clearance proceeding Aβ pathology [8]. However, further studies are needed to determine if AQP4 changes precede protein aggregation and neurodegeneration.

Furthermore, AQP4 emerges as an additional marker to the traditional glial fibrillary acidic protein (GFAP) which shows promise as a blood astrocytic injury marker. The limitations of GFAP as a differential diagnostic marker and its poor protein stability in CSF [9] emphasize the need for additional biomarkers to assess astrocytic status. Additionally, there is no fluid biomarker to address the status of the blood–brain barrier (BBB). AQP4 could be a potential biomarker for BBB impairment due to the high astrocyte expression surrounding the blood vessels and the required astrocytic polarity for the right BBB permeability. However, further analyses are needed to test this hypothesis.

The cross-sectional design of the study poses certain constraints, emphasizing the need for longitudinal assessments to evaluate the temporal dynamics of AQP4 as a progression marker.

In conclusion, our study highlights AQP4 as a promising candidate for AD diagnosis and underscores its potential as a biomarker in AD. These findings advocate for further research to ascertain the diagnostic potential and the temporal occurrence of increased CSF AQP4 levels in the AD-continuum. In addition, future studies are needed to determine the possible use of CSF AQP4 as an objective readout of therapeutic effects in clinical trials and the use of AQP4 as a fluid biomarker for BBB damage. Furthermore, AQP4 protein levels could help determine the role of astrocytes and the glymphatic system in AD.

## Supplementary Information

**Additional file 1**. **Supplementary Materials and Methods**. **Table S1.** Demographic and clinical characterization of discovery cohort from Ulm University Hospital. **Table S2.** Demographic and clinical characterization of validation cohorts (cohort I and cohort II). **Table S3**. Diagnosis of the control participants included in the discovery and validation cohorts. **Table S4**. Repeatability and intermediate precision of AQP4 ELISA using three CSF samples with high, medium, and low levels of AQP4 (QC1, QC2, and QC3). **Table S5**. Spike-recovery of AQP4 ELISA in three CSF samples. **Table S6**. Summary of the multivariable regression model in the AQP4 discovery cohort. **Table S7**. Correlation of CSF AQP4 with MMSE. **Figure S1.** Analytical validation of the AQP4 ELISA. **Figure S2.** Correlation between CSF AQP4 levels obtained by ELISA and antibody-based suspension bead array with different AQP4 antibodies. **Figure 3.** Frequency distribution of CSF AQP4 levels in the discovery cohort, validation cohort I, and validation cohort II with the calibration curves. **Figure S4.** Visualization of the multivariable regression model with diagnosis as independent variable and age as covariate. **Figure 5**. Correlation of CSF AQP4 with t-tau, p-tau in AD patients and all diagnostic groups in the discovery cohort, Cohort I and Cohort II.

## Data Availability

All data generated or analysed during this study are included in this published article and its supplementary information.

## Abbreviations

AD: Alzheimer’s disease
ALS: Amyotrophic lateral sclerosis
AQP4: Aquaporin 4
BBB: Blood–brain-barrier
CSF: Cerebrospinal fluid
LBD: Lewy body disease
MCI: Mild cognitive impairment
