# Supplementary material for: Aquaporin-4 as a cerebrospinal fluid biomarker of Alzheimer’s disease
Source: Transl Neurodegener. 2024 Nov 29;13:57. doi: 10.1186/s40035-024-00451-8 (PMC11606142; doi:10.1186/s40035-024-00451-8)
Supplement: Supplementary file 1 — Additional file 1. Supplementary Materials and Methods. Table S1. Demographic and clinical characterization of discovery cohort from Ulm University Hospital. Table S2. Demographic and clinical characterization of validation cohorts (cohort I and cohort II). Table S3. Diagnosis of the control participants included in the discovery and validation cohorts. Table S4. Repeatability and intermediate precision of AQP4 ELISA using three CSF samples with high, medium, and low levels of AQP4 (QC1, QC2, and QC3). Table S5. Spike-recovery of AQP4 ELISA in three CSF samples. Table S6. Summary of the multivariable regression model in the AQP4 discovery cohort. Table S7. Correlation of CSF AQP4 with MMSE. Figure S1. Analytical validation of the AQP4 ELISA. Figure S2. Correlation between CSF AQP4 levels obtained by ELISA and antibody-based suspension bead array with different AQP4 antibodies. Figure 3. Frequency distribution of CSF AQP4 levels in the discovery cohort, validation cohort I, and validation cohort II with the calibration curves. Figure S4. Visualization of the multivariable regression model with diagnosis as independent variable and age as covariate. Figure 5. Correlation of CSF AQP4 with t-tau, p-tau in AD patients and all diagnostic groups in the discovery cohort, Cohort I and Cohort II. [file 40035_2024_451_MOESM1_ESM.pdf]

Supplementary Materials and Methods

Human participants and clinical characterization

Discovery cohort: Ulm University Hospital

The discovery cohort was collected at the Neurology department of Ulm University Hospital, Germany, between 2010 and 2022. The cohort included 157 participants from the following diagnostic groups: 40 Alzheimer's disease (AD), 21 primary progressive aphasia (PPA), 20 behavioral variant frontotemporal dementia (bvFTD), 17 amyotrophic lateral sclerosis (ALS), 21 Lewy body disease (LBD) and 38 controls (CON) (Supplementary Table 1). Patients were diagnosed according to international guidelines for disease diagnosis [1-6]. AD patients were checked for AD core biomarkers. The cut-off values for AD core biomarkers measured with Lumipulse® G600-II system (Fujirebio Europe, Ghent, Belgium) were 0.07, 400, and 60 pg/ml for Aβ42/40, t-tau and p-tau, respectively. Moreover, the cut-offs used for ELISA (INNOTEST®, Fujirebio) measurements were 550, 300, and 62 pg/ml for amyloid-β 1-42 peptide (Aβ42), t-tau and p-tau, respectively. The CON participants were analyzed for any sign of neuroinflammation or neurodegeneration by CSF analysis of cell count, total protein, lactate, immunoglobulins quotient, albumin quotient (Qalb), and oligoclonal IgG bands. Furthermore, only individuals who did not exhibit any pathological findings in their cranial magnetic resonance imaging were included in the study (Supplementary Table 3). The CON presented no significantly age difference with the rest of diagnostic groups, with an age ranging from 55 to 88 years.

Validation Cohort I: University of Perugia

Validation Cohort I consisted of CSF samples collected at the Neurology department of the University of Perugia, Italy, between 2016 to 2023. This cohort included 108 participants: 14 preclinical AD (preAD), 28 AD with mild cognitive impairment (MCI), 29 AD with dementia (ADD) patients and 37 CON (Supplementary Table 2). AD patients underwent a comprehensive neuropsychological evaluation, lumbar puncture, and brain imaging as part of the diagnostic pipeline at baseline. The final AD diagnosis was based on the CSF AD core biomarker profile. The cut-offs used were 0.069, 404 pg/ml, and 56.5 for Aβ42/40, t-tau, and p-tau, respectively. Based on the neuropsychological evaluation and the clinical dementia rating (CDR) scores, the AD patients were classified into three groups: (1) pre-AD patients with a subjective cognitive complaint who did not meet the criteria for MCI; (2) AD-MCI, patients with a clinical dementia rating (CDR) score of 0.5; and (3) ADD patients with a CDR score of ≥ 1.0. All CON participants were subjected to a lumbar puncture to rule out a neurodegenerative condition and presented normal values of AD core biomarkers. Fourteen CON participants reported a subjective cognitive decline but did not meet the MCI criteria. Eleven CON participants were affected by non-degenerative neurological conditions (Supplementary Table 3). None of the CON participants showed cognitive impairment after at least 2 years of follow-up. No age difference was observed

between the different diagnostic groups, except AD-MCI which were significantly younger ($p = 0.022$) than CON.

Cohort II: University of Turin

Cohort II was collected at the Aging Brain and Memory Clinic of the Department of Neuroscience of the University Hospital “Città della Salute e della Scienza” of Turin, Italy. All the 87 patients were recruited consecutively among those who underwent lumbar puncture between the 1st of April 2021 and the 1st of April 2023. The cohort comprises 33 patients with AD, including 23 at MCI stage and 10 at the dementia stage. Additionally, it comprises 17 MCI patients with suspected non-Alzheimer pathology (non-AD MCI) (Supplementary Table 2). The MCI patients with suspected non-Alzheimer pathology include 1 patient fulfilling the diagnostic criteria for normal-pressure hydrocephalus. The control group consisted of 18 cognitively unimpaired individuals (Supplementary Table 3). All those 18 individuals showed values of Aβ42/40 ratio within normal limits. The cut-offs used were 0.063, 450 pg/ml, and 60 for Aβ42/40, t-tau, and p-tau, respectively. The diagnoses of AD and suspected non-Alzheimer pathology were made according to Jack et al [7] and the other most recent internationally approved criteria for neurocognitive disorders [2, 3, 8-12]. Patients with MCI were distinguished from demented patients according to Alberts et al [13]. MCI patients showed a significant cognitive impairment at the neuropsychological examination, but no significant impairment at Activities of Daily living (ADL) [14] and Instrumental Activities of Daily Living (IADL) [15] questionnaires. No statistically significant age differences were observed between the different diagnostic groups.

The studies obtained approval from respective ethics committees (Ulm University Hospital, No. 20/10; University Perugia, 19369/08/AV registry no: 1287/08 date: 9 October 2008 and University of Turin, 0114576) and adhered to the most recent iteration of the Declaration of Helsinki. Prior to their participation, all the participants or their caregivers received a thorough explanation of the study's procedures and gave written consent. The sample size was based on availability

Sample collection and biomarker measurements

CSF was obtained through lumbar puncture and centrifuged between 2000-2500 g for 10 minutes at 4ºC. The resulting supernatants were aliquoted and promptly stored at -80ºC no later than 2 hours after collection. CSF AD core biomarkers (Aβ42, amyloidβ 1-40 peptide (Aβ40), p-tau, and t-tau) were analyzed with Lumipulse® G600-II system (Fujirebio Europe, Ghent, Belgium) or ELISA (INNOTEST®, Fujirebio). Stratified randomization was performed based on the clinical diagnosis, and samples were analyzed in a blinded manner. All CSF used for analytical validation and as QC samples were remnant samples of healthy donors from Ulm University Hospital. Samples were thawed at room temperature (RT), vortexed, centrifuged at 10,000 g for 5 min at 4°C, and kept on ice until measurement.

AQP4 ELISA protocol

Wells of a 96-well plate (F96 Nunc Immunoplate MaxiSorp, Thermo Fischer Scientific) were coated with 100 µl of 3.3 µg/ml capture antibody (Cell signaling, 59678BF) in coating buffer (100 mM bicarbonate-carbonate buffer pH 9.6) to be adsorbed overnight at 4ºC. The next day, the antibody solution was discarded, and 300 µl of blocking buffer (1% goat serum in PBS 0.05% Tween) were added for 2h at RT. After discarding the blocking buffer, 100 µl of the sample were added and incubated for 1.5 h at 20ºC. The sample solution was discarded, wells were washed 3 times with 300 µl of washing buffer (PBS with 0.05% Tween), and 100µl of 0.66 µg/ml biotinylated detector antibody (Abcam, ab248213) in blocking buffer were applied for 1h at 20ºC. One hour later, the last washing step was conducted, and 100 µL 3,3',5,5'-Tetramethylbenzidin (TMB) (ThermoScientific, 34028) were added to each well and incubated for 30 minutes at RT. The reaction was stopped with 100µL 1M HCl per well. The optical density (OD) was measured at 450nm against a reference wavelength of 570nm with an ELISA reader (Epoch2, BioTek). A four-parameter logistic model (without weighting factor) was fitted to the calibration curve.

Antibody-based suspension bead array assay

Two polyclonal were selected from the Human Protein Atlas project (www.proteinatlas.org) (HPA014784 and HPA014944) and two from other providers (Abnova, PAB20767 and Proteintech, 16473-1-AP). The antibodies were conjugated onto uniquely color-coded magnetic carboxylated microspheres (MagPlex, Luminex Corporation) after activation of the carboxylic surface with EDC-NHS. Each bead identity corresponded to one antibody. After quenching the remaining bead activity, beads were pooled together into a suspension bead array.

In parallel, CSF samples were labeled with biotin, as previously described [16]. Samples were further diluted 1/8 and incubated overnight with the antibody-coupled beads. Antigen targets were cross-linked to the antibody-coated beads using paraformaldehyde. A streptavidin-conjugated fluorophore (Streptavidin R-phycoerythrin conjugate, Invitrogen, diluted to 0.2 µg/ml in PBS 0.05% Tween) was added for the detection of the captured proteins. The Flex-map 3D instrument (Luminex Corporation) was employed for the readout, with median fluorescence intensity (MFI) per bead ID per sample serving as the measurement parameter.

Statistical analyses

Data processing, analysis, and visualization were conducted using R (version 4.2.2, R Foundation for Statistical Computing) and GraphPad Prism (version 8.3.0, La Jolla, California, USA). R packages dplyr, tidyverse, ggplot2, ggpubr, ggbeeswarm, emmeans, dunn.test, and pROC were employed. Multiple linear regression models, incorporating age, and sex as covariates, assessed the impact of clinical diagnosis on CSF AQP4 concentrations. A stepwise backward elimination method identified key

variables. Assumptions of linearity, homoscedasticity, and normality of residuals were verified for model validity. To address skewed and non-normally distributed biomarker concentration data, a logarithmic transformation was applied before conducting further analysis. Tukey's test facilitated pairwise comparisons of estimated marginal means. Univariate analysis involved Shapiro-Wilk and Levene's tests for normality and variance homogeneity. Bonferroni correction was employed for multiple testing. Two-sided hypothesis testing was applied throughout the analyses. Non-parametric Spearman's correlation were analyzed. Receiver operating characteristics (ROC) curves illustrated sensitivity and specificity at various cut-off points. Significance levels were denoted as *p < 0.05, **p < 0.01, and ***p < 0.001.

ELISA analytical validation procedure

The ELISA assay was analytically validated for CSF applications following the previously described guidelines [17]. The following parameters were analyzed: limits of quantification, precision, parallelism, spike-recovery, dilutional linearity, sample stability, and specificity. Sixteen blanks, consisting of the blocking buffer used for each respective assay, were included to calculate the lower limit of quantification (LLOQ).

$$LLOQ = mean + 10 * SD$$

Three CSF samples from single control patients or from pooling different remnants from health donors were used for the analytical testing. For repeatability (intra-assay) and intermediate-precision (inter-assay), three CSF samples were aliquoted and measured in 5 replicates on 3 non-consecutive days. The precision was expressed in terms of the coefficient of variation (CV%) [17], with a tolerance of 15% for repeatability and 20% for intermediate precision. For parallelism, three CSF samples were serially diluted (two-fold) in blocking buffer over the calibrator range. Both neat and diluted samples were measured in duplicate. The optimal minimum required dilution (MRD) was the sample's lowest dilution, allowing a sample recovery between 75% and 125% for the following dilutions [18].To assess spike-recovery, CSF samples were diluted to the final dilution used in the assay (MRD or higher dilution), spiked with low, medium, and high concentrations of calibrator antigen (all within the linear range of the calibration curve), and measured in duplicate. Spiked recombinant protein consisted of less than 10% of the total sample volume to avoid further dilution of the sample. 20% variation was accepted for spike-recovery.

$$\text{Spike} - \text{recovery}(\%) = \frac{\text{measured concentration spiked sample}}{\text{measured concentration neat sample} + \text{ theoretical concentration spiked}} \text{ x } 100$$

Moreover, for dilutional linearity, samples were spiked at a concentration 90x above the highest calibration point and diluted 2.5 and 2-fold in blocking buffer to a concentration within the working range. Subsequently, measurements were conducted in duplicate, with an acceptable 15% variation. Protein stability was tested in two CSF samples from control patients. The samples were aliquoted and subjected to 1 to 5 freeze-thaw (FT) cycles or stored under different conditions: at 4ºC for 24h, 3 days, and 5 days; or at RT for 2h, 4h, 24h, 3 days, and 5 days. In all cases, protein stability was determined by comparing the protein concentration to a reference sample stored at -80ºC. Last, to exclude any cross-reactivity with human serum albumin (HSA) and IgG, a physiological and higher concentration of HSA (200 µg/ml and 600 µg/ml) (Sigma, A8763-250MG) and IgG (30 µg/ml and 90 µg/ml) (Sigma, I4506-10MG) was added both in blank and CSF. Moreover, to assess further cross-reactivity with other proteins, an alignment between the epitope of the antibodies (if provided) and the human proteome was performed in NCBI-BLAST ® (https://blast.ncbi.nlm.nih.gov/) [19].

ELISA analytical validation results

Repeatability and intermediate precision were assessed for three QC samples with high, medium, and low levels of AQP4, ranging from 2.6% to 4.5% and 12.9% to 16.3%, respectively (Supplementary Table 4). The specified acceptance criteria (75-125%) for parallelism were successfully fulfilled, with a minimum required dilution of 1/4 (Supplementary Figure 1A). Moreover, all subsequent dilutions remained within the acceptable range, demonstrating parallelism throughout the experiment. Furthermore, the binding characteristics of the antibodies to the endogenous analyte in the biological matrix closely resembled those observed for the recombinant protein in the blocking buffer (Supplementary Figure 1B). Moreover, the criteria were effectively met for spike-recovery (recovery percentage 95-107%) (Supplementary Table 5) and dilutional linearity (Supplementary Figure 1C). Last, AQP4 was stable for up to five freeze-thaw cycles and when stored at 4ºC and RT for up to five days (Supplementary Figure 1D-F). The assay detected AQP4 in CSF samples with an lower limit of quantification LLOQ of 127 pg/ml.

To add additional evidence to the correct target binding, we employed a previously published method [20, 21] and developed a multiplexed antibody-based suspension bead array assay using four distinct AQP4 antibodies (HPA014784, HPA014944, PAB20767 and 16473-1-AP). The data derived from the bead array and the ELISA described a strong correlation for all antibodies, thereby supporting the specificity of the ELISA method (Supplementary Figure 2). Additionally, to further assess the assay's specificity, CSF samples were spiked with the most abundant proteins in CSF (HSA and IgGs) [22]. No cross-reactivity between the antibodies used in the assay and HSA or IgG was observed.

To further analyze off-target antibody binding, a pair-wise sequence alignment was performed between the immunogen sequence of the capture antibody (ab248213) and the human proteome using the NCBI-BLAST ® algorithm [19]. No protein with highly aligned protein sequences was found. The exact epitope of the detector antibody (59678BF) is unknown and the pair-wise sequence alignment for this antibody could not be performed.

Performance of AQP4 ELISA in Cohort Measurements

Each cohort was processed on a single day, with samples analysed on parallel ELISA plates. In addition to the clinical samples, three QC samples were measured in triplicate on each plate with a CV% ranging from 2.7% to 18.0%. All AQP4 CSF concentrations were above the LLOQ and fell within the calibrator range (62.5-3500 pg/ml), with the exception of five samples. One sample (cohort II) was below the LLOQ (88 pg/ml), while four (3x discovery cohort and 1x validation cohort I) exceeded the upper limit of the calibration curve and their concentrations were extrapolated (**Supplementary Figure 3**). Furthermore, all calibrator points demonstrated acceptable precision below 20%, (62.5 pg/ml), with a back-calculated concentration ranged from 96% to 115%, except for the lowest calibrator which is below LLOQ.

In both validation cohorts, no age differences were observed between the clinical groups, except for the AD-MCI patients of validation cohort I, which were younger than the CON (P = 0.022). Moreover, the covariates analyzed (age and sex) were not significant in the models

Supplementary Tables

Table S1. Demographic and clinical characterization of discovery cohort from Ulm University Hospital

		CON	AD	PPA[c]	bvFTD	ALS	LBD	Total
**Demographic features**	**Sample size**	38	40	21	20	17	21	157
	**Sex (F/M)**	21/17	21/19	8/13	6/14	9/8	8/13	73/84
	**Age (years)**	69 (63-74)	74 (67-79)	68 (60-74)	62 (60-67)	62 (48-72)	71 (67-75)	69 (61-75)
**Cognitive scores**	**MMSE**	NA	23 (18-26)	23 (18-25)	27 (23-28)	NA	NA	23 (19-27)
	**CDR-SOB**	NA	3.5 (2.0-5.5)	2.0 (1.0-3.5)	3.0 (2.0-4.0)	NA	NA	2.8 (2.0-4.5)
**AD core biomarkers**	**Aβ42 (pg/ml)**	1266 (873-1493) [a]	373 (287-527) [b]	635 (518 - 1053)	926 (763-1148)	NA	NA	707 (433-1088)
	**Aβ40 (pg/ml)**	13031 (8574-14363) [a]	10237 (7920-12860) [b]	10775 (8749-12128)	10104 (8210-13899)	NA	NA	10806 (8546-13286)
	**Aβ42/40**	0.10 (0.10-0.10) [a]	0.04 (0.03-0.04) [b]	0.07 (0.05-0.10)	0.10 (0.09-0.11)	NA	NA	0.08 (0.04-0.10)
	**t-tau (pg/ml)**	275 (223-346) [a]	692 (500-1220) [b]	455 (328-639)	306 (225-368)	NA	NA	407 (278-675)
	**p-tau (pg/ml)**	37 (30-41) [a]	103 (76-186) [b]	62 (36-101)	38 (27-49)	NA	NA	51 (35-100)
	**A+T+ (%)**	0	97.5	42.8	10	NA	NA	41.2

Age, MMSE, CDR-SOB, Aβ42, Aβ40, Aβ42/40, t-tau, and p-tau are displayed as median and IQR in brackets. Only AD core biomarkers measured with Lumipulse ® are displayed in the table. [a, b] 27 CON and 20 AD were analyzed with the ELISA INNOTEST ® and their values are therefore not included in the table. [C] Within the PPA group, 7 patients presented logopenic variant, 7 semantic variant and 7 non-fluent variant PPA. AD, Alzheimer's disease; ALS, amyotrophic lateral sclerosis; bvFTD, behavioral variant frontotemporal dementia; CDR-SOB, clinical dementia rating-sum of boxes; CON, control; F, female; IQR, interquartile range; LBD, Lewy body disease; M, male; MMSE, mini-mental state examination; NA, not available; PPA, primary progressive aphasia.

Table S2. Demographic and clinical characterization of validation cohorts (cohort I and cohort II)

Cohort I: University of Perugia						
	**Diagnostic group**	**CON**	**preAD**	**AD-MCI**	**ADD**	**Total**
**Demographic features**	**Sample size**	37	14	28	29	108
	**Sex (F/M)**	19/18	11/3	17/11	20/9	67/41
	**Age (years)**	75 (72-78)	76 (69-76)	73 (69-75)	76 (70-79)	74 (71-77)
**Cognitive scores**	**MMSE**	27 (23-28)	27.5 (27-28)	24 (21-26)	16 (11-21)	24 (19-27)
	**CDR**	0.5 (0-0.5)	0 (0-0.5)	0.5 (0.5-0.5)	1 (1-2)	0.5 (0.5-1)
**AD core biomarkers**	**Aβ42 (pg/ml)**	1011 (753-1355)	647 (565-832)	633 (489-786)	537 (409-822)	724 (538-1009)
	**Aβ40 (pg/ml)**	10815 (8329-12923)	14399 (9262-15958)	14453 (10198-16904)	11953 (8181-15408)	11850 (8946-15679)
	**Aβ42/40**	0.097 (0.092-0.106)	0.056 (0.049-0.063)	0.045 (0.040-0.060)	0.049 (0.046-0.058)	0.06 (0.047-0.093)
	**t-tau (pg/ml)**	260 (205-313)	432 (341-639)	705 (543-898)	731 (576-1006)	536 (294-765)
	**p-tau (pg/ml)**	34 (28-43)	69 (59-100)	90 (71-119)	91 (70-128)	69 (40-100)
	**A+T+ (%)**	0	100	100	100	65.7
**Cohort II: University of Turin**						
	**Diagnostic group**	**CON**	**AD-MCI**	**ADD**	**non-AD MCI**	**Total**
**Demographic features**	**Sample size**	18	23	10	17	68
	**Sex (F/M)**	8/10	15/8	8/2	9/8	40/28
	**Age (years)**	68 (62-75)	73 (69-74)	72 (62-77)	70 (63-74)	71 (65-75)
**Cognitive scores**	**MMSE**	28 (27-29)	22 (21-24)	16 (7-23)	26 (24-28)	25 (21-27)
**AD core biomarkers**	**Aβ42 (pg/ml)**	987 (654-1384)	463 (294-523)	317 (231-394)	548 (396-972)	508 (327-743)
	**Aβ40 (pg/ml)**	9846 (7035-13383)	10289 (7410-11186)	7726 (6870-8563)	6526 (4187-9202)	8209 (6198-11109)
	**Aβ42/40**	0.098 (0.090-0.108)	0.043 (0.038-0.048)	0.039 (0.033-0.052)	0.091 (0.078-0.104)	0.069 (0.042-0.096)
	**t-tau (pg/ml)**	192 (79-274)	795 (532-1004)	740 (432-1357)	319 (174-512)	414 (202-797)
	**p-tau (pg/ml)**	28 (21-40)	118 (74-184)	128 (82-190)	38 (19-103)	66 (30-138)
	**A+T+ (%)**	0	100	90	0	47

Age, MMSE, CDR, Aβ42, Aβ40, Aβ42/40, t-tau, and p-tau are displayed as median and IQR in brackets. AD, Alzheimer's disease; ADD, Alzheimer’s disease dementia; CDR, clinical dementia rating; CON, control; F, female; IQR, interquartile range; M, male; MCI, mild cognitive impairment; MMSE, mini-mental state examination; preAD, preclinical AD.

Table S3. Diagnosis of the control participants included in the discovery and validation cohorts.

Diagnosis	n	Cohort
Abducens nerve paresis	2	Discovery cohort Ulm
Brainstem transient ischemic attack	1	
Catatonia	1	
Epilepsy	2	
Facial palsy	4	
Gait disorder, subcortical vascular encephalopathy	1	
Headache	5	
Headache, subcortical vascular encephalopathy	1	
Intracranial hypertension, depression	1	
Mandibular arthrosis	1	
Migraine	1	
Myopathy	1	
Neuralgic shoulder amyotrophy	1	
Polyneuropathy	2	
Post-zoster neuralgia	1	
Ptosis	1	
Seizures	1	
Temporal arteritis (Giant cell arteritis)	3	
Transient ischemic attack	1	
Psychiatric disorder	1	
Trochlear palsy	3	
Vestibular neuritis	2	
Vitamin B12 deficiency	1	
Bipolar disorder	1	Validation Cohort I Perugia
Cranial neuropathy	1	
Headache	1	
Late onset epilepsy	4	
Melanoma metastasis and epilepsy	1	
Minimal cognitive impairment	3	
Multifactorial MCI	2	
Multifocal ischemic stroke	1	
Non-neurodegenerative amnesic syndrome	1	
Normal pressure hydrocephalus	3	
Paraneoplastic anti-HU polyneuropathy	1	
Psychiatric disorder	3	
Stable MCI	1	
Subjective cognitive decline	6	
Transient global amnesia	1	
Vascular dementia	3	
Vascular MCI	4	
Autoimmune encephalitis	1	Validation Cohort II Turin
Myelitis	1	
Neurologically healthy patient in spinal anaesthesia	4	
Polyneuropathy	4	
Subjective cognitive decline	8	

Table S4. Repeatability and intermediate precision of AQP4 ELISA using three CSF samples with high, medium, and low levels of AQP4 (QC1, QC2, and QC3).

S_r = repeatability standard deviation, S_{RW} = intermediate precision standard deviation

Reference sample	AQP4 concentration (pg/mL)	S_r (pg/ml)	Repeatability (CV_r%)	S_{Rw} (pg/ml)	Intermediate precision (CV_{Rw}%)
**QC1**	6526.1	169.5	2.6	1063.1	16.3
**QC2**	3245.4	128.3	4.0	417.9	12.9
**QC3**	1739.0	77.9	4.5	239.3	13.8
**Mean**			**3.7**		**14.3**

Table S5. Spike-recovery of AQP4 ELISA in three CSF samples.

	Recovery percentage (%)		
**Sample**	**Low spike (300 pg/mL)**	**Medium spike (800 pg/mL)**	**High spike (1500 pg/mL)**
**Sample 1**	111	99	96
**Sample 2**	104	105	95
**Sample 3**	106	97	94
**Mean recovery percentage (%)**	**107**	**100**	**95**

Table S6. Summary of the multivariable regression model in the AQP4 discovery cohort.

	Estimate	Std.Error	t value	P-value
**Intercept**	7.613750	0.204414	37.247	< 2e-16
**Diagnosis AD**	0.405436	0.080954	5.008	1.52e-06
**Diagnosis PPA**	0.238612	0.096669	2.468	0.0147
**Diagnosis bvFTD**	0.135728	0.099853	1.359	0.1761
**Diagnosis ALS**	0.043447	0.107757	0.403	0.6874
**Diagnosis LBD**	0.080369	0.096636	0.832	0.4069
**Age (years)**	0.012913	0.002847	4.536	1.16e-05

Data was log-transformed to reduce skewness and bring closer to a normal distribution. Residual standard error: 0.3553 on 150 degrees of freedom. Multiple R-squared: 0.3054, Adjusted R-squared: 0.2776. F-statistic: 10.99 on 6 and 150 DF, p-value: 3.91e-10

Table S7. Correlation of CSF AQP4 with MMSE

	Pilot cohort	Validation cohort I	Validation cohort II
Spearman r (95%C)I	-0.1 (-0.33 - 0.13), p=0.35	-0.14 (-0.33 - 0.06), p=0.14	-0.48 (-0.65 - -0.27), p<0.0001

Supplementary Figures

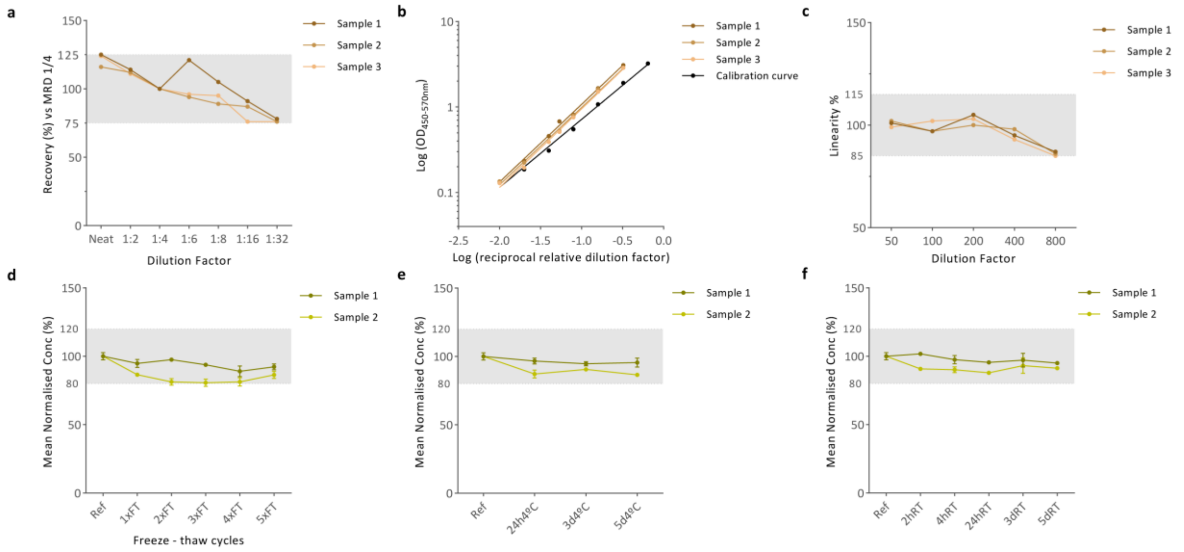

Fig. S1. Analytical validation of the AQP4 ELISA. (**a**) Recovery (%) for each dilution of three CSF samples using 1/4 as the MRD. Recovery (%) was calculated as the ratio between the measured analyte concentration * DF and the measured MRD concentration * DF * 100. (**b**) Parallelism of three CSF samples serially diluted within the quantifiable range of the calibration curve. (**c**) Dilutional linearity of three CSF samples spiked with a high concentration of AQP4 and diluted two-fold into the calibration curve range. Mean percent linearity was calculated as (observed concentration at dilution X) * (DFX) / (observed concentration at dilution X-1) * (DFX-1) * 100. (**d**) Protein stability of AQP4 after multiple FT cycles. The samples were thawed and kept at RT for 4 hours before being frozen at -80°C until analysis. (**e**) Protein stability of AQP4 after keeping the samples at 4°C for 24 hours, 3 days, and 5 days. (**f**) Protein stability of AQP4 after keeping the samples at RT for 2 hours, 4 hours, 24 hours, 3 days, and 5 days until analysis. The horizontal dashed lines represent the accepted variation range. AQP4, Aquaporin 4; CSF, cerebrospinal fluid; DF, dilution factor; FT, freeze-thaw; MRD, minimum required dilution; OD, optical density; Ref, reference; RT, room temperature.

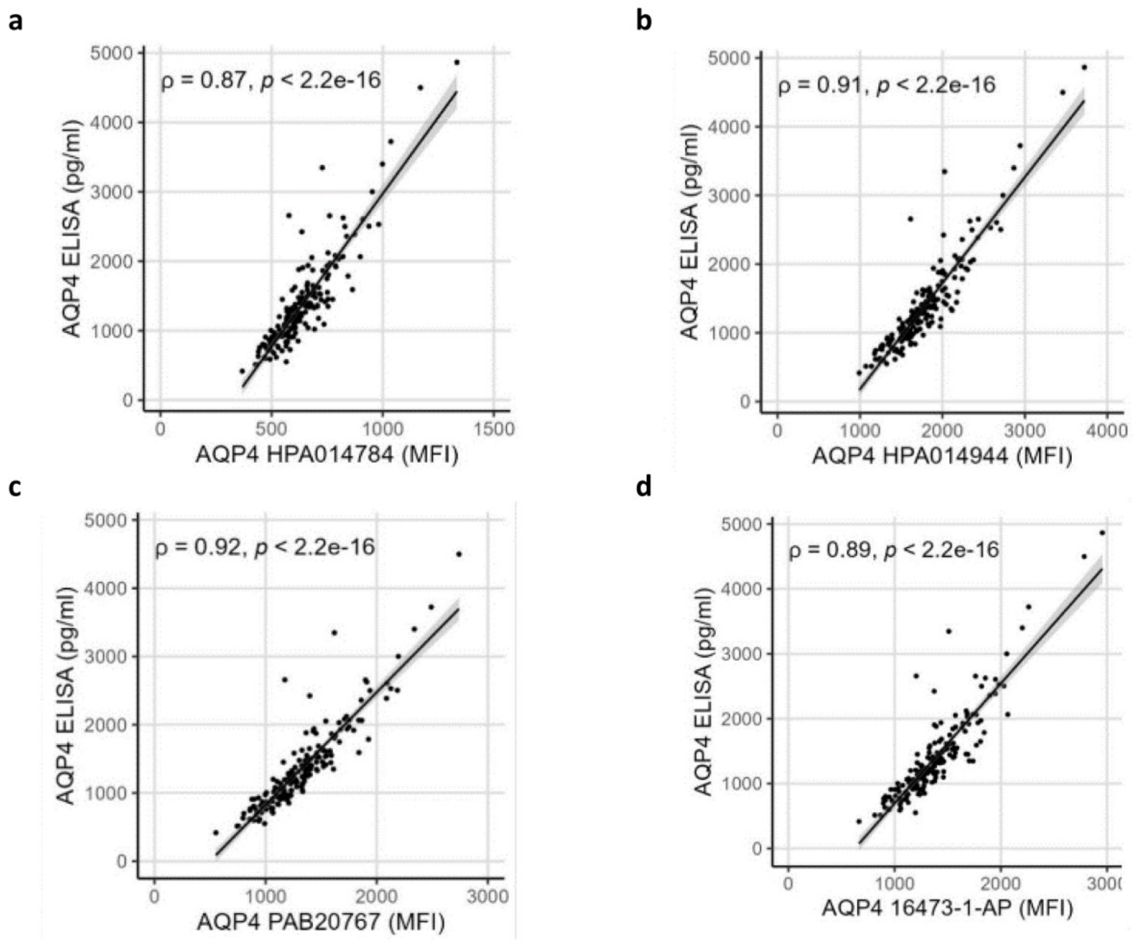

Fig. S2. Correlation between CSF AQP4 levels obtained by ELISA and antibody-based suspension bead array with different AQP4 antibodies: (**a**) HPA014784, (**b**) HPA014944, (**c**) PAB20767 and (**d**) 16473-1-AP in a cohort of 180 participants. Correlation analyses were computed using the Spearman rank correlation coefficient (ρ). The grey area represents the confidence interval. AQP4, Aquaporin-4; CSF, cerebrospinal fluid; MFI, median fluorescent intensity.

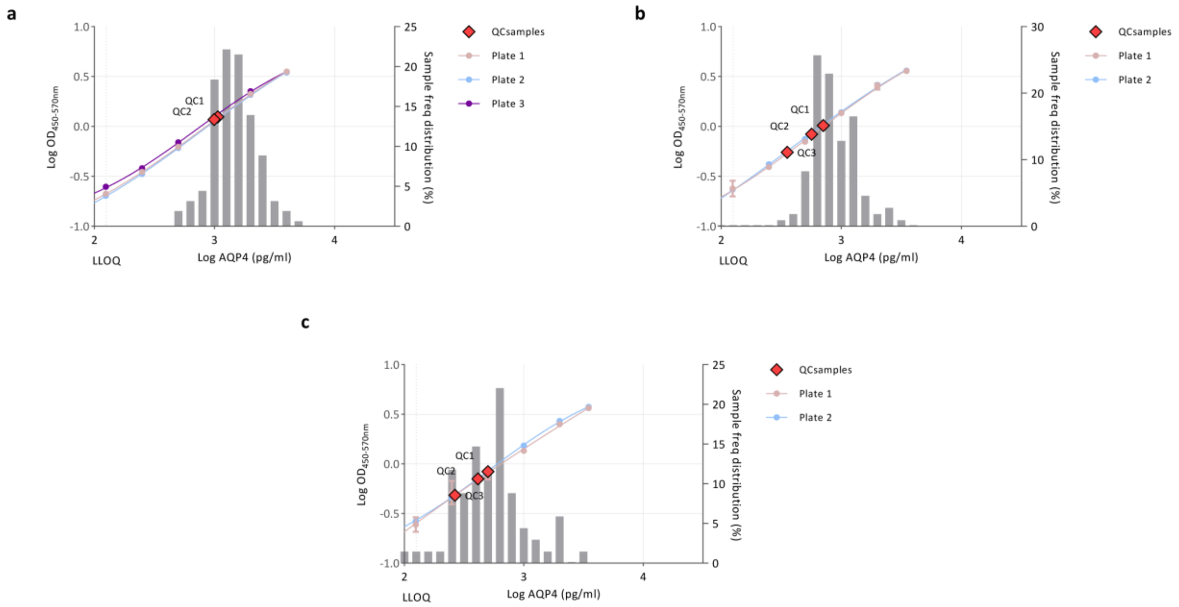

Fig. S3. Frequency distribution of CSF AQP4 levels in the discovery cohort (n = 157) (**a**), validation cohort I (n = 108) (**b**), and validation cohort II (n = 68) (**c**) with the calibration curves. Three samples of the discovery cohort and one sample from the cohort I exceeded the calibration curve limit, and their concentrations were extrapolated. One sample from the cohort II was bellow LLOQ. The CSF concentrations of QC samples used in the cohorts are highlighted in red. The vertical dashed line represents the LLOQ. AQP4, aquaporin-4; CSF, cerebrospinal fluid; LLOQ, lower limit of quantification; OD, optical density; QC, quality control.

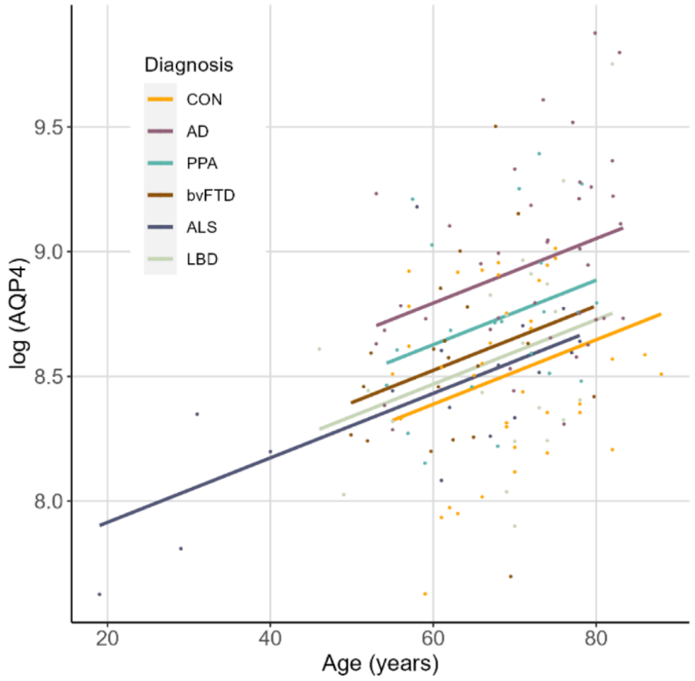

Fig. S4. Visualization of the multivariable regression model with diagnosis as independent variable and age as covariate. $Y = \beta CON + \beta Diagnosis(Diagnosis) + Xage\, \beta age + \varepsilon$. AD, Alzheimer's disease; ALS; amyotrophic lateral sclerosis; AQP4, Aquaporin-4; bvFTD, behavioral variant frontotemporal dementia; CON, control; LBD, Lewy body disease; PPA, primary progressive aphasia.

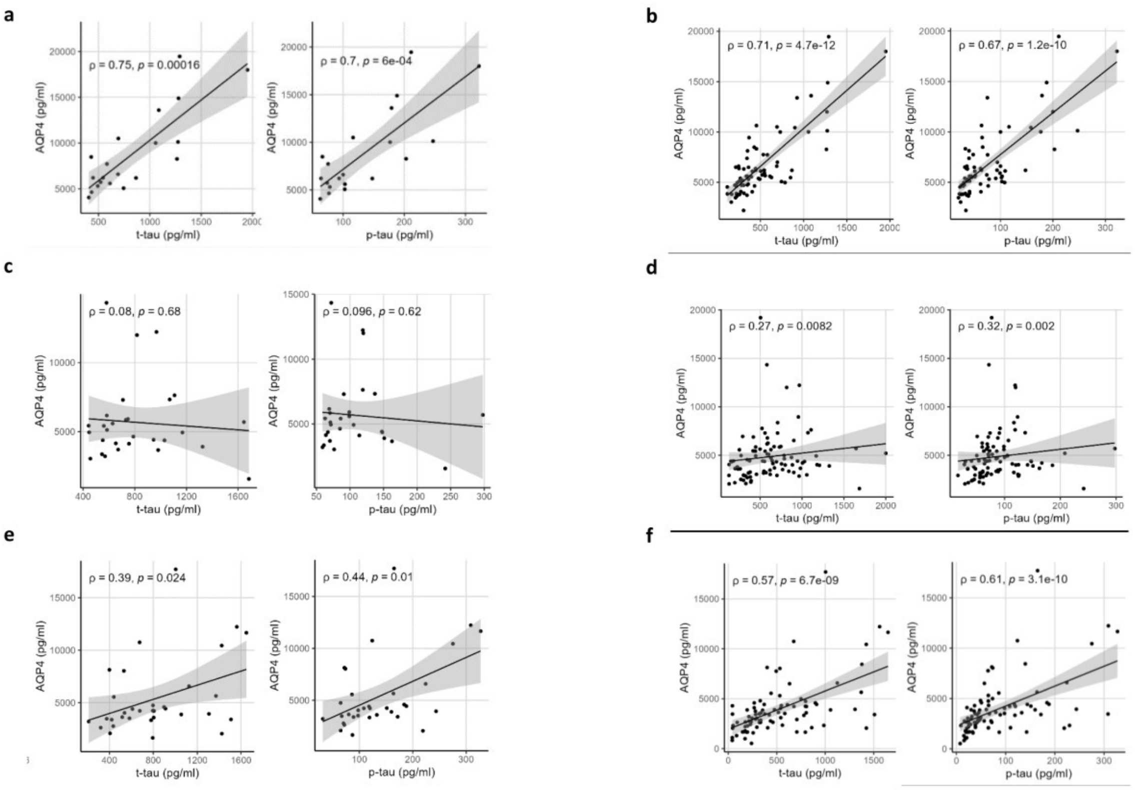

Fig. S5. Correlation of CSF AQP4 with t-tau, p-tau in AD patients (**a**, **c**, **e**) and all diagnostic groups (**b**, **d**, **f**) in the discovery cohort (**a**, **b**), Cohort I (**c**, **d**) and Cohort II (**e**, **f**). Only samples measured with Lumipulse ® were included. Correlation analyses were computed using the Spearman rank correlation coefficient (ρ). The grey area represents the confidence interval. AQP4, aquaporin-4; CSF, cerebrospinal fluid.

References

1. Dubois B, Feldman HH, Jacova C, Hampel H, Molinuevo JL, Blennow K, et al. Advancing research diagnostic criteria for Alzheimer's disease: the IWG-2 criteria. The Lancet Neurology. 2014;13(6):614-29.
2. Rascovsky K, Hodges JR, Knopman D, Mendez MF, Kramer JH, Neuhaus J, et al. Sensitivity of revised diagnostic criteria for the behavioural variant of frontotemporal dementia. Brain : a journal of neurology. 2011;134(Pt 9):2456-77.
3. Gorno-Tempini ML, Hillis AE, Weintraub S, Kertesz A, Mendez M, Cappa SF, et al. Classification of primary progressive aphasia and its variants. Neurology. 2011;76(11):1006-14.
4. Emre M, Aarsland D, Brown R, Burn DJ, Duyckaerts C, Mizuno Y, et al. Clinical diagnostic criteria for dementia associated with Parkinson's disease. Movement disorders : official journal of the Movement Disorder Society. 2007;22(12):1689-707; quiz 837.
5. Brooks BR, Miller RG, Swash M, Munsat TL. El Escorial revisited: revised criteria for the diagnosis of amyotrophic lateral sclerosis. Amyotrophic lateral sclerosis and other motor neuron disorders : official publication of the World Federation of Neurology, Research Group on Motor Neuron Diseases. 2000;1(5):293-9.
6. Hughes AJ, Daniel SE, Kilford L, Lees AJ. Accuracy of clinical diagnosis of idiopathic Parkinson's disease: a clinico-pathological study of 100 cases. Journal of neurology, neurosurgery, and psychiatry. 1992;55(3):181-4.
7. Jack CR, Jr., Bennett DA, Blennow K, Carrillo MC, Dunn B, Haeberlein SB, et al. NIA-AA Research Framework: Toward a biological definition of Alzheimer's disease. Alzheimer's & dementia : the journal of the Alzheimer's Association. 2018;14(4):535-62.
8. Charidimou A, Boulouis G, Frosch MP, Baron JC, Pasi M, Albucher JF, et al. The Boston criteria version 2.0 for cerebral amyloid angiopathy: a multicentre, retrospective, MRI-neuropathology diagnostic accuracy study. The Lancet Neurology. 2022;21(8):714-25.
9. McKeith IG, Boeve BF, Dickson DW, Halliday G, Taylor JP, Weintraub D, et al. Diagnosis and management of dementia with Lewy bodies: Fourth consensus report of the DLB Consortium. Neurology. 2017;89(1):88-100.
10. Hoglinger GU, Respondek G, Stamelou M, Kurz C, Josephs KA, Lang AE, et al. Clinical diagnosis of progressive supranuclear palsy: The movement disorder society criteria. Movement disorders : official journal of the Movement Disorder Society. 2017;32(6):853-64.
11. Armstrong MJ, Litvan I, Lang AE, Bak TH, Bhatia KP, Borroni B, et al. Criteria for the diagnosis of corticobasal degeneration. Neurology. 2013;80(5):496-503.
12. Relkin N, Marmarou A, Klinge P, Bergsneider M, Black PM. Diagnosing idiopathic normal-pressure hydrocephalus. Neurosurgery. 2005;57(3 Suppl):S4-16; discussion ii-v.
13. Albert MS, DeKosky ST, Dickson D, Dubois B, Feldman HH, Fox NC, et al. The diagnosis of mild cognitive impairment due to Alzheimer's disease: recommendations from the National Institute on Aging-Alzheimer's Association workgroups on diagnostic guidelines for Alzheimer's disease. Alzheimer's & dementia : the journal of the Alzheimer's Association. 2011;7(3):270-9.
14. Katz S, Ford AB, Moskowitz RW, Jackson BA, Jaffe MW. Studies of Illness in the Aged. The Index of Adl: A Standardized Measure of Biological and Psychosocial Function. Jama. 1963;185:914-9.
15. Lawton MP, Brody EM. Assessment of older people: self-maintaining and instrumental activities of daily living. The Gerontologist. 1969;9(3):179-86.
16. Haggmark-Manberg A, Nilsson P, Schwenk JM. Neuroproteomic Profiling of Cerebrospinal Fluid (CSF) by Multiplexed Affinity Arrays. Methods Mol Biol. 2017;1598:247-54.
17. Andreasson U, Perret-Liaudet A, van Waalwijk van Doorn LJ, Blennow K, Chiasserini D, Engelborghs S, et al. A Practical Guide to Immunoassay Method Validation. Frontiers in neurology. 2015;6:179.
18. Valentin MA, Ma S, Zhao A, Legay F, Avrameas A. Validation of immunoassay for protein biomarkers: bioanalytical study plan implementation to support pre-clinical and clinical studies. Journal of pharmaceutical and biomedical analysis. 2011;55(5):869-77.
19. Altschul SF, Gish W, Miller W, Myers EW, Lipman DJ. Basic local alignment search tool. Journal of molecular biology. 1990;215(3):403-10.
20. Bergstrom S, Oijerstedt L, Remnestal J, Olofsson J, Ullgren A, Seelaar H, et al. A panel of CSF proteins separates genetic frontotemporal dementia from presymptomatic mutation carriers: a GENFI study. Molecular neurodegeneration. 2021;16(1):79.

21. Bergstrom S, Remnestal J, Yousef J, Olofsson J, Markaki I, Carvalho S, et al. Multi-cohort profiling reveals elevated CSF levels of brain-enriched proteins in Alzheimer's disease. Annals of clinical and translational neurology. 2021;8(7):1456-70.
22. Zhang J. Proteomics of human cerebrospinal fluid - the good, the bad, and the ugly. Proteomics Clinical applications. 2007;1(8):805-19.
